# Deaths from Chloroform

**Published:** 1872-01

**Authors:** 


DEATHS FROM CHLOROFORM.—According to the *Boston Medical and Surgical Journal*, seventy deaths from chloroform were reported in England previous to 1870. Twenty-nine cases, collected from American and foreign journals, have been noted since that date; nineteen cases in the issue of June 15, 1871, and ten cases in that of December 14, 1871.

